# Increased Dkk‐1 plasma levels may discriminate disease subtypes in myeloproliferative neoplasms

**DOI:** 10.1111/jcmm.13753

**Published:** 2018-07-05

**Authors:** Cristina Mambet, Laura Necula, Simona Mihai, Lilia Matei, Coralia Bleotu, Mihaela Chivu‐Economescu, Oana Stanca, Aurelia Tatic, Nicoleta Berbec, Cristiana Tanase, Carmen C. Diaconu

**Affiliations:** ^1^ MyeloAL Program Stefan S Nicolau Institute of Virology Bucharest Romania; ^2^ Biochemistry‐Proteomics Laboratory “Victor Babes” National Institute of Pathology Bucharest Romania; ^3^ Department of Hematology Coltea Clinical Hospital Bucharest Romania; ^4^ Carol Davila University of Medicine and Pharmacy Bucharest Romania; ^5^ Center of Hematology and Bone Marrow Transplantation Fundeni Clinical Institute Bucharest Romania

**Keywords:** circulating biomarker, cytokine array, Dickkopf‐related protein 1, myeloproliferative neoplasms

## Abstract

Alterations in the bone marrow niche induced by abnormal production of cytokines and other soluble factors have been associated with disease progression in classical BCR‐ABL1 negative myeloproliferative neoplasms (MPN). Variations in circulating proteins might reflect local disease processes and plasma proteome profiling could serve to identify possible diagnostic and prognostic biomarkers. We employed a human cytokine array to screen for 105 distinct analytes in pooled plasma samples obtained from untreated young MPN patients (<35 years) with different clinical phenotypes and driver mutations, as well as from healthy individuals. Among molecules that exhibited significantly increased levels in MPN patients versus controls, the top of the list was represented by Dickkopf‐related protein 1 (Dkk‐1), which also showed the highest potential for discrimination between MPN subtypes. In the next step, a quantitative ELISA was used to measure plasma Dkk‐1 levels in 30 young‐onset MPN—10 essential thrombocythemia (ET), 10 polycythemia vera (PV), 10 pre‐fibrotic primary myelofibrosis (pre‐PMF)—and 10 controls. The results suggested that plasma Dkk‐1 levels could differentiate ET from pre‐PMF, in *JAK2* V617F‐positive as well as in *CALR*‐positive patients, and also ET from PV in *JAK2* V617F‐positive patients.

## 1 INTRODUCTION

Philadelphia‐negative classical myeloproliferative neoplasms (MPNs) represented by polycythemia vera (PV), essential thrombocythemia (ET) and primary myelofibrosis (PMF) are clonal disorders that emerge in the haematopoietic stem cell compartment (HSC). They are defined by an excessive production of functional mature blood cells from one or more myeloid lineages.^1^

Three classes of somatic activating driver mutations localized within specific exons of Janus Kinase 2 (*JAK2*), thrombopoietin receptor (*MPL/TPOR*) and calreticulin (*CALR*) genes are key players in the majority of MPNs^2^ leading to an improper activation of JAK2 signalling.^3^ The single HSC that harbours the oncogenic mutation—MPN stem cell—has to acquire a selective advantage over its normal counterpart to ensure the clonal expansion needed for the development of an MPN phenotype. Strong evidence indicates that the expanded neoplastic clone progressively impairs the bone marrow microenvironment creating a favourable malignant niche.^4^ This process is mediated by a aberrant production of cytokines such as tumour necrosis factor‐α (TNF‐α), transforming growth factor‐β (TGF‐β), platelet‐derived growth factor (PDGF), basic fibroblast growth factor (FGF‐b), hepatocyte growth factor (HGF), interleukin‐8 (IL‐8), vascular endothelial growth factor (VEGF), oncostatin M (OSM)^5^ and/or interleukin‐1β (IL‐1β).^6^ The alterations induced in the bone marrow niche, namely neural damage, angiogenesis, expansion of abnormal osteolineage cells and reticulin fibrosis, have been associated with MPN progression.^6^ Both malignant and non‐malignant cells of haematopoietic system are involved in the abnormal cytokine production via activation of JAK‐STAT3 pathway.^7^ Plasma/serum profiles of various cytokines and other soluble proteins might reflect local disease processes and could serve as noninvasive diagnostic or prognostic tools in patients with myeloproliferative disorders.^8^

In this study, we used a human cytokine array to measure plasma levels of multiple proteins in young MPN patients (<35 years) with different clinical phenotypes and driver mutations. Our aim was to identify possible biomarkers for discriminating between different MPN subtypes.

## 2 MATERIAL AND METHODS

### 2.1 Patients and samples

The study was approved by the local ethics committee, and research was conducted according to the World Medical Association Declaration of Helsinki. We used platelet‐poor EDTA plasma samples and genomic DNA (gDNA) isolated from peripheral blood granulocytes previously obtained by standard procedure from 30 MPN patients and stored in the biobank of Stefan S Nicolau Institute of Virology, Romania. Patients were referred from the Hematology Services of “Coltea” and “Fundeni” Clinic Romanian Hospitals to the Institute of Virology for detection of *JAK2 *V617 F mutation by allele‐specific PCR. In *JAK2* V617F‐negative MPN patients, we analysed *CALR* exon 9 mutations using gDNA samples and a previously described protocol for direct Sanger sequencing.^9^

Clinical data were provided for each case. All the patients gave an informed consent at the time of blood collection. For comparing results, we also included 10 age‐matched healthy controls.

### 2.2 Real‐time quantitative PCR (qPCR) for *JAK2* V617F mutational load quantification

For the quantification of *JAK2* V617F mutation in gDNA from granulocytes, we used the ipsogen JAK2 MutaQuant Kit (Qiagen) with a measured limit of detection of 0.1% following the protocol recommended by the manufacturer for Applied Biosystems 7300 Real‐Time PCR instrument (Thermo Fisher Scientific). The copy number of mutant and wild‐type *JAK2* alleles was calculated from the standard curves using SDS software version 1.4.1 (Thermo Fisher Scientific). *JAK2* V617F allele burden was expressed as the percentage of *JAK2* V617F copy number relative to the total number of *JAK2* copies (wild‐type plus mutant).

### 2.3 Human cytokine array

As a first testing approach, we used Proteome Profiler Human XL Cytokine Array Kit (ARY022B, R&D Systems, Abingdon, UK) for semiquantitative determination of cytokines, chemokines, growth factors, angiogenesis markers and other soluble proteins in plasma samples of MPN patients and controls. The multianalyte assay contains nitrocellulose membranes, each being spotted in duplicate with capture antibodies to 105 distinct analytes and also with control antibodies. The protocol recommended by the manufacturer was followed. Briefly, each membrane was incubated with 100 μL of undiluted plasma sample at 4°C overnight. After extensive washing to remove unbound materials, the membranes were incubated with biotinylated detection antibodies. Subsequently, streptavidin‐HRP was added followed by chemiluminescent detection reagents. The chemiluminescent signals generated at each spot corresponding to the amount of protein bound were recorded by the MicroChemi 4.2 system (DNR Bio‐Imaging Systems, Israel) after membrane exposure. The intensity of signals (pixel densities) was quantified with ImageJ 1.42 (National Institute of Health, Bethesda, MD, USA). For each analyte, the average signal of the duplicate spots was calculated, corrected for background signal and normalized to the average signal of the membrane reference spots.

### 2.4 Quantitative ELISA for the measurement of Dickkopf‐related protein 1 (Dkk‐1) plasma levels

Dkk‐1 levels in plasma samples from MPN patients and controls were measured by quantitative solid‐phase ELISA (DKK100B, Quantikine ELISA Human Dkk‐1 Immunoassay, R&D Systems, Abingdon, UK) according to manufacturer's protocol. Each plasma sample was diluted 1:4 using Calibrator Diluent RD5‐24 and tested in duplicate. Final DKK‐1 concentrations were obtained by interpolation from the standard curve and multiplication with the dilution factor.

### 2.5 Statistical analysis

Statistical tests were performed with GraphPad Prism 6.01 for Windows. To compare the fold change in analyte expression in MPN patients versus control, one‐way ANOVA test was employed for multiple groups and Student's unpaired t test for two groups. Differences in Dkk‐1 levels among MPN subtypes and controls were analysed by nonparametric Kruskal‐Wallis test H followed by Mann‐Whitney U test for one‐to‐one comparison. Also, Kruskal‐Wallis test H was used to assess the differences in *JAK2* V617F allele burden among ET, PV and pre‐PMF patients. Correlation between plasma levels of Dkk‐1 and *JAK2* V617F allele burden was evaluated using the nonparametric Spearman rank correlation coefficient. *P* values <.05 were considered statistically significant.

## 3 RESULTS

### 3.1 Patient characteristics and plasma pool preparation

To avoid bias from co‐morbidities and treatment, we selected 30 untreated patients that received a diagnosis of MPN at young age (<35 years): 10 ET, 10 PV and 10 pre‐PMF patients. *JAK2* V617F mutation was present in 20 patients (66.6%). The other 10 patients harboured *CALR* mutations, classified in type 1 (del52, n = 6) and type 2 (ins5, n = 4) mutations.^9^ The diagnosis of each MPN subtype was established according to the World Health Organization (WHO) 2016 criteria.^10^

For the initial proteomic screening, we prepared 5 pools of plasma samples from patients sharing the same MPN subtype and driver mutation and 1 pool from controls (10 age‐matched subjects with normal blood counts). Clinical characteristics, haematological parameters, type of driver mutation and also *JAK2* V617F allele burden of MPN patients corresponding to each pool are summarized in Table 1.

**Table 1:** Clinical characteristics, haematological parameters, type of driver mutation and *JAK2* V617F allele burden of MPN patients corresponding to each pool analysed in cytokine array

Patient no.	Sample pool no.	MPN subtype	Age	Gender	Hb g/dL	WBC count × 10^9^/L	PLT count × 10^9^/L	Driver mutation	Allele burden (%)
1		ET	24	M	14.5	7.5	780	*JAK2* V617F	17.9
2		ET	26	F	12.8	6.9	725	*JAK2* V617F	17.0
3	**I**	ET	24	F	13.0	7.7	638	*JAK2* V617F	37.0
4		ET	28	F	14.7	7.9	852	*JAK2* V617F	28.7
5		ET	26	M	15.4	10.9	1128	*JAK2* V617F	20.4
6		ET	25	F	12.0	6.2	1000	*CALR* type 1	
7		ET	29	F	11.8	9.8	1247	*CALR* type 2	
8	**II**	ET	24	M	16	7.7	750	*CALR* type 2	
9		ET	32	M	14	8.8	736	*CALR* type 2	
10		ET	33	F	13.4	6.9	903	*CALR* type 2	
11		PV	28	F	15.6	9.0	721	*JAK2* V617F	28.2
12		PV	34	F	16.8	12.0	677	*JAK2* V617F	62.5
13		PV	35	F	15.8	11.3	1010	*JAK2* V617F	31.1
14		PV	30	F	14.1	6.1	594	*JAK2* V617F	28.2
15	**III**	PV	23	F	17.8	8.2	308	*JAK2* V617F	81.6
16		PV	27	F	13.8	4.6	588	*JAK2* V617F	42.1
17		PV	25	M	17	12.0	860	*JAK2* V617F	19.1
18		PV	35	F	15	7.9	500	*JAK2* V617F	27.7
19		PV	31	F	16.8	9.5	797	*JAK2* V617F	28.4
20		PV	35	M	16.3	13.8	967	*JAK2* V617F	17.4
21		pre‐PMF	33	F	11.6	14	720	*JAK2* V617F	24.8
22		pre‐PMF	35	F	13.8	9.9	763	*JAK2* V617F	23.7
23	**IV**	pre‐PMF	30	F	13.9	17.8	620	*JAK2* V617F	64.9
24		pre‐PMF	33	F	13.5	8.9	389	*JAK2* V617F	18.8
25		pre‐PMF	26	F	13.9	9.4	560	*JAK2* V617F	21.2
26		pre‐PMF	35	M	12.2	27.3	898	*CALR* type 1	
27		pre‐PMF	33	M	12.2	10.9	922	*CALR* type 1	
28	**V**	pre‐PMF	32	F	12.1	10.3	650	*CALR* type 1	
29		pre‐PMF	29	F	11.9	18.2	955	*CALR* type 1	
30		pre‐PMF	35	M	12.1	19	713	*CALR* type 1	

PV, polycythemia vera; ET, essential thrombocythemia; pre‐PMF, pre‐fibrotic primary myelofibrosis; Hb, haemoglobin; WBC, white blood cells, PLT, platelet.

### 3.2 Relevant plasma proteins detected in MPN patients by the human cytokine array

The preliminary multianalyte screening for the differentially expressed cytokines among various MPN patients and controls was performed in 6 plasma pools (5 from MPN patients and 1 from controls), and 6 individual plasma specimens from each pool, using 12 array membranes.

**Figure 1:**
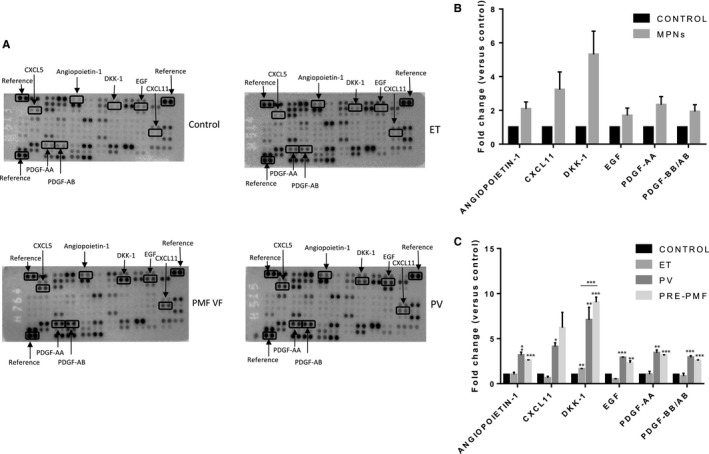
Plasma protein profiling by cytokine array in MPN subtypes and controls. A, Representative images of array membranes corresponding to different MPN subtypes and control. The spots marked in boxes present differences in signal intensity among array membranes. Reference spots were used for signal normalization. B, Expression level of proteins that exhibited at least 1.5 fold change in MPN patients (compared to controls). In cytokine array, protein expression was assessed by densitometric analysis. The mean value of pixel densities in controls was set at 1.0, and the fold change was calculated for each protein. Data are presented as mean fold change ± standard error of the mean (SEM). C, Fold change in protein expression in MPN subtypes (compared to controls). The mean value of pixel densities in controls was set at 1.0, and the fold change was calculated for each protein, in each MPN subtype, in three independent experiments. Data are presented as mean fold change ± standard error of the mean (SEM). *P < .05, **P < .01, ***P < .001, Student's unpaired *t* test

Examples of array membranes displaying visual differences in signal intensity are shown in Figure 1A.

After completion of chemiluminescent signal measurements and statistical analysis, 6 proteins were identified as having at least 1.5‐fold change in expression in MPN patients versus controls: angiopoietin‐1, Dkk‐1, EGF (epidermal growth factor), I‐TAC (interferon‐inducible T cell alpha chemoattractant, CXCL11), PDGF‐AA and PDGF‐AB/BB (Figure 1B).

Among analytes that exhibited a significant fold increase in MPN patients versus controls, Dkk‐1 showed the highest potential for discrimination between MPN subtypes and was selected for further study (*P* = .0031, one‐way ANOVA, Figure 1C).

### 3.3 Plasma levels of Dkk‐1 in MPN subtypes

Levels of Dkk‐1 were measured by quantitative ELISA in the plasma samples of 30 young MPN patients and 10 healthy controls that were previously tested with the cytokine array.

Subsequently, the concentrations of Dkk‐1 calculated from the standard curve were normalized to platelet count, as platelets represent a major source for the circulating Dkk‐1,^11^ and MPN patients exhibit thrombocytosis.

**Figure 2:**
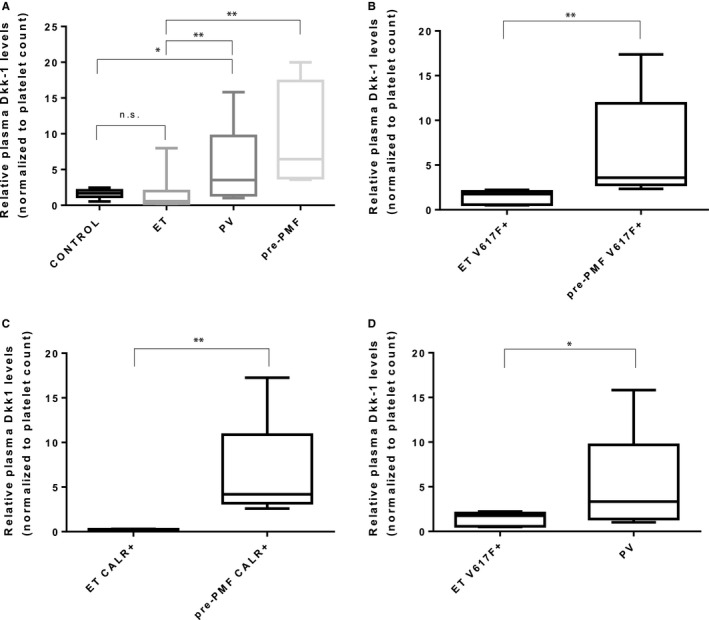
Relative plasma Dkk‐1 levels (normalized to platelet count) in MPN subtypes and controls. Differences in plasma Dkk‐1 levels across all MPN subtypes and controls (A), between ET and pre‐PMF in *JAK2* V617F‐positive patients (B), between ET and pre‐PMF in *CALR*‐positive patients (C), and between ET and PV in *JAK2* V617F‐positive patients (D). Box plots (median, 25%‐75% interquartile range, minimum and maximum values) of normalized plasma Dkk‐1 levels are shown. *P < .05, **P < .01, Mann–Whitney *U* test

Normalized plasma Dkk‐1 levels were different among MPN subtypes and controls (*P* = .0005; Kruskal–Wallis *H* test). PV patients, as well as pre‐PMF patients, had significantly higher levels of circulating Dkk‐1 compared to controls (*P* = .041, respectively, *P* = .0001), while no significant difference in Dkk‐1 levels was observed between ET patients and controls (*P* = .183) (Figure 2A). Taking into account the MPN subtypes, pre‐PMF patients displayed increased levels of plasma Dkk‐1 versus ET patients (*P* = .0012, Figure 2A). The discriminative power of plasma Dkk‐1 concentrations was maintained when *JAK2* V617F‐positive and *CALR*‐positive patients were analysed separately (*P* = .0079, respectively, *P* = .008, Figure 2B,C). Additionally, plasma Dkk‐1 levels were higher in PV patients versus *JAK2* V617F‐positive ET patients (*P* = .0451, Figure 2D).

### 3.4 Distribution of *JAK2* V617F allele burden among MPN subtypes and correlation analysis between plasma levels of Dkk‐1 and *JAK2* V617F allele burden

In case of *JAK2* V617F‐positive patients, the median value of mutated allele burden was calculated separately for ET, PV and pre‐PMF: 20.4%, 28.3%, and 23.7%, respectively. No statistically significant difference in V617F allele burden was found among MPN subtypes (*P* = .369; Kruskal–Wallis *H* test). Also, we observed no correlation between plasma levels of Dkk‐1 and *JAK2* V617F allele burden (Spearman correlation coefficient *r* = .066, *P* = .800).

## 4 DISCUSSION

Our findings suggest that plasma Dkk‐1 levels could discriminate ET from pre‐PMF, in both *JAK2* V617F‐positive and mutant *CALR*‐positive patients.

The WHO 2016 classification of myeloid neoplasms recognizes pre‐PMF as a separate MPN subgroup.^10^ From a clinical perspective, pre‐PMF patients often exhibit isolated thrombocytosis at disease onset, similar to ET patients. Although they share a similar incidence of major thrombotic events, pre‐PMF patients pose a higher risk to develop complications, such as progression to overt PMF and leukaemic transformation, as well as a higher mortality rate. Therefore, the differentiation between the two MPN phenotypes is very useful in practice. At the moment, this is based mainly on bone marrow histology.^12^, ^13^

Secondly, we found that circulating levels of Dkk‐1 might distinguish also ET from PV in *JAK2* V617F‐positive patients. In our group of young MPN patients, prodromal or “masked” PV (mPV) predominated. As shown previously, the distinction between mPV and ET exhibits both prognostic and therapeutic relevance, to address the higher incidence of thrombosis in PV versus ET and the need of phlebotomies in PV for obtaining a haematocrit less than 45%.^14^ Even when applying the new WHO 2016 thresholds for haemoglobin and haematocrit, it was found that in 14% of cases the PV, the diagnosis was missed. Thus, serum erythropoietin levels and bone marrow biopsy are mandatory for establishing a diagnosis of PV in these cases.^15^

The involvement of Dickkopf family members Dkk‐1/2/3/4, in MPNs, was previously documented in the case of Dkk‐3. Medinger et al^16^ reported increased circulating Dkk‐3 levels in PV that have been related to an enhanced platelet activation, with possible relevance for the pathogenesis of thrombotic events. Dkk‐1/2/3/4 are soluble inhibitors of Wnt signalling and could modulate also other signalling pathways.^17^ To our knowledge, there are no studies concerning the association of Dkk‐1 with MPNs.

Dkk‐1 plays an important role during embryogenesis and it was involved in bone homoeostasis in adulthood.^18^ From a pathological perspective, it was suggested that Dkk‐1 released by platelets and endothelial cells might be involved in the inflammatory reactions within the atherosclerotic plaques.^11^ As a potent inhibitor of canonical Wnt signalling—a pathway aberrantly activated in many types of cancer—Dkk‐1 was initially considered a tumour suppressor^19^ However, many studies have found that Dkk‐1 is overexpressed in malignant tissue and/or serum of patients with various solid cancers (breast, cervical, endometrial, oesophageal, gastric, colorectal, liver, urothelial, lung, etc) being associated with tumour growth, angiogenesis and unfavourable prognosis.^19^ Also, increased Dkk‐1 levels in bone marrow plasma and blood plasma of multiple myeloma patients were correlated with the presence of osteolytic lesions.^20^ Interestingly, in a recent study, it was shown that exosomes produced by acute myeloid leukaemia cell lines and patient‐derived blasts stimulated the expression of Dkk‐1 in bone marrow stromal cells that contributed to the transformation of normal haematopoietic niche into a leukaemia cell supportive microenvironment.^21^

In our study, significantly higher plasma Dkk‐1 levels in PV and pre‐PMF compared to ET patients could be related to bone marrow niche alterations, in particular to angiogenesis. This finding is in line with previous reports showing an increased bone marrow angiogenesis—assessed by microvessel density (MVD)—in MPNs (PMF > PV > ET). Moreover, an increased MVD was observed even in the pre‐fibrotic stage of PMF.^22^, ^23^, ^24^ The source of Dkk‐1 in MPNs could be represented by megakaryocytes and/or bone marrow stromal cells.

A particular finding of this study was that *JAK2* V617F allele burden did not show statistically significant differences among MPN subtypes. These could be a feature of the analysed group consisting of young MPN patients, as in a recent study, it was reported that adolescents and young adults with PV and PMF displayed significantly lower JAK2 V617F allele burdens compared to older patients having a similar disease phenotype.^25^ Accordingly, Dkk‐1 levels in plasma were not correlated with the mutated allele load suggesting that they might reflect early changes in bone marrow microenvironment rather than a direct relation with clonal burden.

Thus, plasma DKK‐1 levels could represent a potential noninvasive biomarker for differentiation between MPN subtypes, especially in young patients that often display an overlap in clinical onset of disease.

## CONFLICT OF INTEREST

The authors confirm that there are no conflict of interests.

## AUTHOR CONTRIBUTION

CM, LN, SM, LM and CCD substantially contributed to research design, the acquisition, analysis and interpretation of data, and wrote the manuscript. OS, AT and NB recruited the patients, performed research and analysis of the data. CB, MC‐E and CT analysed data and reviewed the manuscript. CCD designed the study and approved final version.
